# Glycemic Gravity: The Pull of HbA1c on Balanoposthitis Severity

**DOI:** 10.7759/cureus.97151

**Published:** 2025-11-18

**Authors:** Shreya Sharma, Kapila Verma, Radhika Thakur, Vishesh Budhwani, Sapna Gupta

**Affiliations:** 1 Dermatology, LN Medical College and Research Center, Bhopal, IND

**Keywords:** balanoposthitis, candida, correlation analysis, diabetes mellitus, glycemic control, hba1c

## Abstract

Background

Balanoposthitis, an inflammatory condition of the glans penis and foreskin, is common in diabetic patients, especially those with poor glycemic control. Chronic hyperglycemia, as measured by HbA1c, may exacerbate the severity of balanoposthitis. Limited research exists on the direct relationship between HbA1c and disease severity, making this link crucial to understand for better management.

Aim

The primary aim was to evaluate the correlation between HbA1c and balanoposthitis severity in diabetic males. Objectives included assessing severity by HbA1c levels, exploring the impact of glycemic control on symptom progression, and providing clinical insights for treatment improvement.

Materials and methods

This was a cross-sectional study involving a sample size of 50 diabetic male patients diagnosed with balanoposthitis. HbA1c was measured using standard laboratory tests. Disease severity was assessed using a Clinical Severity Scoring System, where symptoms including erythema, edema, discharge, and ulceration were individually scored from 0 (none) to 3 (severe or purulent or multiple deep ulcers). The total severity score ranged from 0 to 24, with a higher score indicating more severe disease. Data analysis involved Pearson’s correlation and regression analysis. Patients were grouped by glycemic control as good (HbA1c ≤ 6%), moderate (HbA1c 7 to 8%), and poor (HbA1c ≥ 9%).

Key findings

The distribution of patients showed that the largest group, 44%, had poor glycemic control (HbA1c ≥ 9%) and presented with severe balanoposthitis (score 13-24). Thirty-six percent of patients had moderate control (HbA1c 7-8%) with moderate balanoposthitis (score 7-12), and 20% had good control (HbA1c ≤ 6%) with mild symptoms (score 0-6). Symptom prevalence and intensity increased significantly with higher HbA1c levels. For patients with poor control (HbA1c ≥ 9%), the prevalence of severe symptoms was high: 90% experienced erythema, 85% experienced itching, edema, and painful burning sensation, and 75% had ulceration. In contrast, patients with good control (HbA1c ≤ 6%) primarily experienced mild symptoms such as itching (30%) and erythema (40%), with ulceration present in only 5%. The statistical analysis showed a strong positive correlation between HbA1c levels and balanoposthitis severity, with a Pearson’s correlation coefficient (r) of 0.45 (p < 0.01). This indicates that as HbA1c increases, severity scores rise significantly. Poor glycemic control was associated with higher severity, including ulceration and pain, whereas good control was linked to mild symptoms.

Conclusions and clinical relevance

HbA1c levels strongly correlate with balanoposthitis severity. Glycemic control is essential for preventing progression to severe symptoms. Early intervention and management of blood glucose can reduce symptom severity and may prevent severe cases. This study highlights the clinical relevance of integrating endocrinology and dermatology, advocating for a holistic approach and collaboration between dermatologists, endocrinologists, and primary care providers for effective management and improved early diagnosis and treatment.

## Introduction

Balanoposthitis is defined as an inflammatory condition involving the glans penis and the foreskin [1-3]. This condition is particularly common in patients with diabetes, especially those who have poor control over their blood glucose levels [4-10]. Chronic hyperglycemia, typically measured by elevated glycated hemoglobin (HbA1c) levels, is hypothesized to exacerbate the severity of balanoposthitis. High glucose levels promote the formation of advanced glycation end-products (AGEs), which in turn drive chronic inflammation and tissue damage, creating a favorable environment for persistent infections and worsening inflammation [11]. However, there is currently limited research that specifically explores the quantitative relationship and correlation between a patient's HbA1c levels and the severity of their balanoposthitis. Establishing and understanding this link is considered crucial for optimizing management and treatment strategies for affected diabetic patients. The aim of this study was to correlate HbA1c with balanoposthitis severity in diabetic males. The primary objective was to evaluate the correlation between HbA1c and balanoposthitis severity. Secondary objectives included assessing severity across different HbA1c levels in diabetic males, exploring the impact of glycemic control on symptom progression, and providing clinical insights for treatment improvement.

## Materials and methods

Study setting, duration, and ethical clearance

This was a cross-sectional observational study conducted at the Dermatology Outpatient Department of LN Medical College and J.K. Hospital, Bhopal, India. The study was carried out over a period of 18 months. The study protocol, including the consent process, was reviewed and approved by the Institutional Ethics Committee or Institutional Review Board (IRB) of the hospital (IRB Letter No.: LNMC&RC/Dean/2023/Ethics/213). All participants provided written informed consent prior to enrollment.

Target population and sampling technique

The target population included adult male patients with a pre-existing diagnosis of diabetes mellitus who presented with clinical signs of balanoposthitis. The study utilized a nonprobability convenience sampling technique to enroll a total sample size of 50 diabetic male patients over the study duration.

Inclusion and exclusion criteria

Inclusion criteria comprised adult males (age ≥ 18 years) with confirmed diabetes mellitus and a clinical diagnosis of balanoposthitis. Exclusion criteria included patients with known nondiabetic causes of balanoposthitis such as drug reactions or fixed drug eruptions, those with immunosuppressive disorders other than diabetes, and those who had received any topical or systemic treatment for balanoposthitis within the preceding two weeks.

Data collection procedure and measurement

Upon enrollment, a detailed history was obtained from each patient, and a comprehensive dermatological examination was performed. Demographic and clinical data, including age, duration of diabetes, and current diabetic medication, were recorded.

HbA1c Measurement

A venous blood sample was collected from each participant, and HbA1c was measured using high-performance liquid chromatography (HPLC), ensuring consistency.

Disease Severity Scoring

Clinical severity of balanoposthitis was quantitatively assessed using a validated 24-point composite clinical severity scale developed by Li et al. in 2021 [12]. This instrument evaluates both subjective symptoms (e.g., pain, pruritus, and dysuria) and objective signs (e.g., erythema, edema, fissuring, and ulceration) on a 0 to 3 scale for each item, yielding a total score ranging from 0 (no disease) to 24 (maximum severity). The scale has demonstrated high interrater reliability (kappa > 0.85) and construct validity in previous dermatologic studies of inflammatory penile conditions. All clinical assessments in the present study were performed by a single resident physician to minimize interobserver variability and ensure consistent application of the validated scoring criteria (Table 1).

**Table 1:** Detailed severity scoring system ¹Lesion area calculated as the percentage of the glans penis surface and inner prepuce with erosion/ulcer. Score = sum of all item values (0-24); 0 indicates no lesion, 24 indicates the most severe condition.

Item	0	1	2	3
Extent of involvement	None	Penis crown	Either the glans or the inner prepuce	Whole glans and inner prepuce
Erythema degree	No	Mildly reddish	Fresh red with edema	Fiery red with apparent edema
Erosion/ulcer area¹	0	1–29%	30–60%	>60%
Number of papules/blisters/pustules	No	1–9	10–30	>30
Smegma	No	Visible smegma upon cotton swabbing	Visible smegma	Dense smegma
Foul odor	No	Slight odor	Easily detected odour	Strong odor
Itching	No	Mild itching	Considerable itching causing irritation	Severe itching even affecting sleep
Pain	No	Mildly painful	Considerable pain causing irritation	Severe pain even affecting sleep

For statistical analysis, patients were stratified into three groups based on their glycemic status: good control (HbA1c ≤ 6% or mild severity, score 0 to 6), moderate control (HbA1c 7 to 8% or moderate severity, score 7 to 12), and poor control (HbA1c ≥ 9% or severe severity, score 13 to 24).

Statistical analysis

Pearson’s correlation coefficient (r) was calculated to determine the strength and direction of the linear relationship between the continuous variable HbA1c and the total severity score. A p-value of <0.05 was considered statistically significant. All statistical and graphical analyses for this study were conducted using Stata software version 17.0 (StataCorp, College Station, Texas).

## Results

Demographic and clinical properties of the sample population

The study enrolled a total sample size of 50 diabetic male patients presenting with balanoposthitis. The mean age of the participants was 35.5 ± 8.2 years, reflecting the typical demographic profile for individuals prone to diabetes-related complications. Clinically, the patients had a mean duration of diabetes of 5.25 ± 3.5 years. These properties provide the clinical context for the observed correlation between glycemic control and disease severity.

Distribution by glycemic control

**Figure 1:**
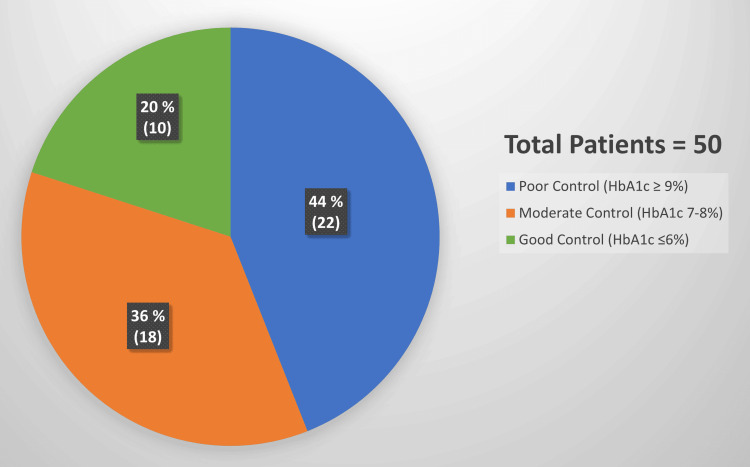
Patient distribution by glycemic control

The sample distribution demonstrated that the largest proportion of patients exhibited poor glycemic control. As shown in Figure 1, 22 patients, representing 44% of the sample (n = 22), were found to have poor glycemic control (HbA1c ≥ 9%) and presented with severe balanoposthitis (score 13 to 24). Eighteen patients (36%, n = 18) had moderate control (HbA1c 7-8%) with moderate balanoposthitis (score 7-12). The remaining 10 patients (20%, n = 10) had good control (HbA1c ≤ 6%) and exhibited mild symptoms (score 0-6).

Symptom prevalence and intensity

The clinical presentation of balanoposthitis correlated directly with the patient's glycemic status. The prevalence and intensity of symptoms increased markedly in the poor control group.

In patients with poor glycemic control (HbA1c ≥ 9%), the most common and severe symptoms included erythema (90% prevalence), itching, edema, and painful burning sensation (all ≥ 85% prevalence). Critically, ulceration was observed in 75% of this group. In contrast, patients with good glycemic control (HbA1c ≤ 6%) predominantly reported mild symptoms such as erythema (40%) and itching (30%), with ulceration present in only 5% of this cohort (Table 2).

**Table 2:** Prevalence and intensity of individual symptoms

Symptom	Mild (≤6%)	Moderate (7-8%)	Severe (≥9%)
Itching	30%	55%	85%
Burning micturition	20%	50%	80%
Erythema	40%	65%	90%
Edema	25%	55%	85%
Painful burning sensation	15%	50%	85%
Ulceration	5%	40%	75%

Correlation analysis

**Figure 2:**
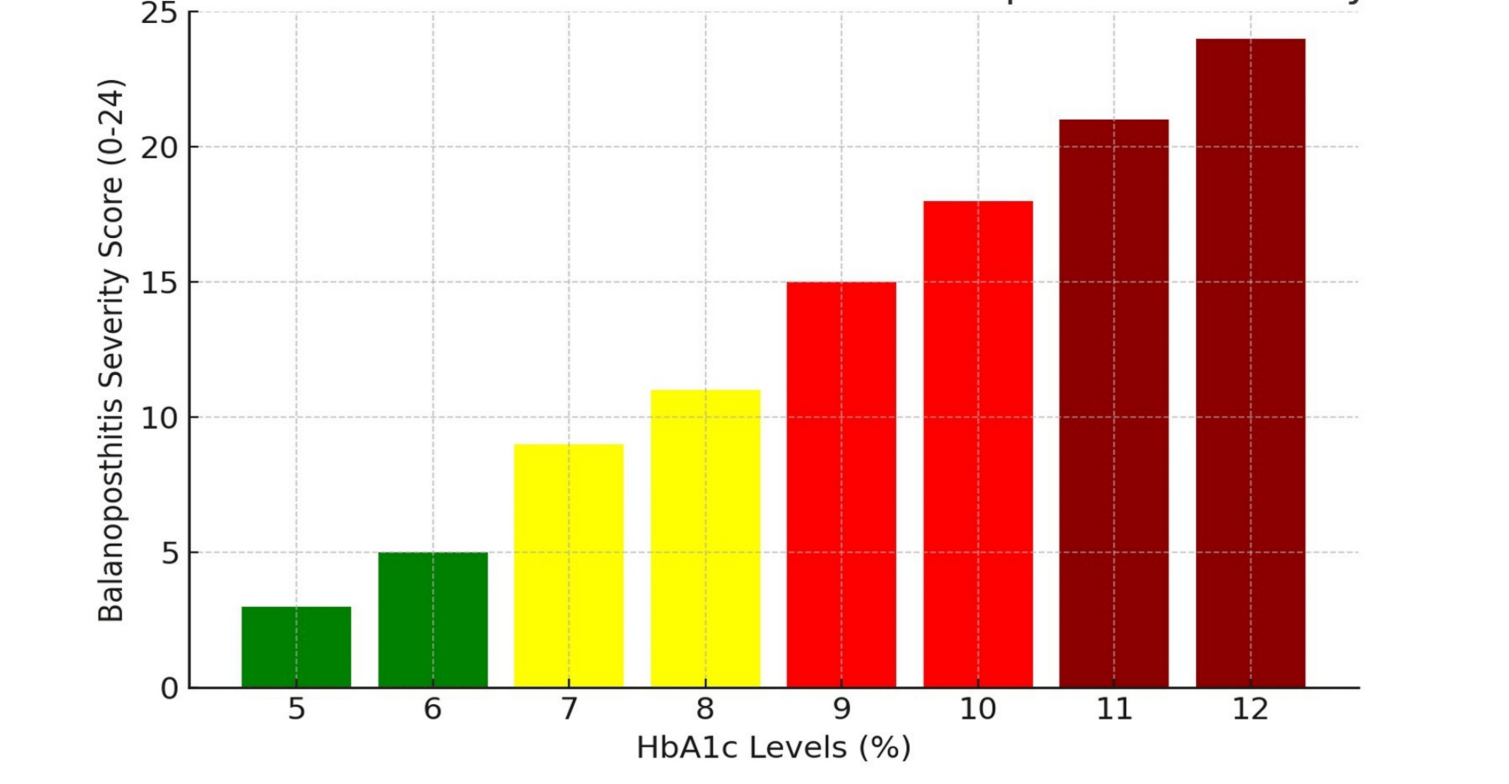
Correlation between HbA1c and balanoposthitis severity Trend: Higher HbA1c linked to higher severity scores.

**Figure 3:**
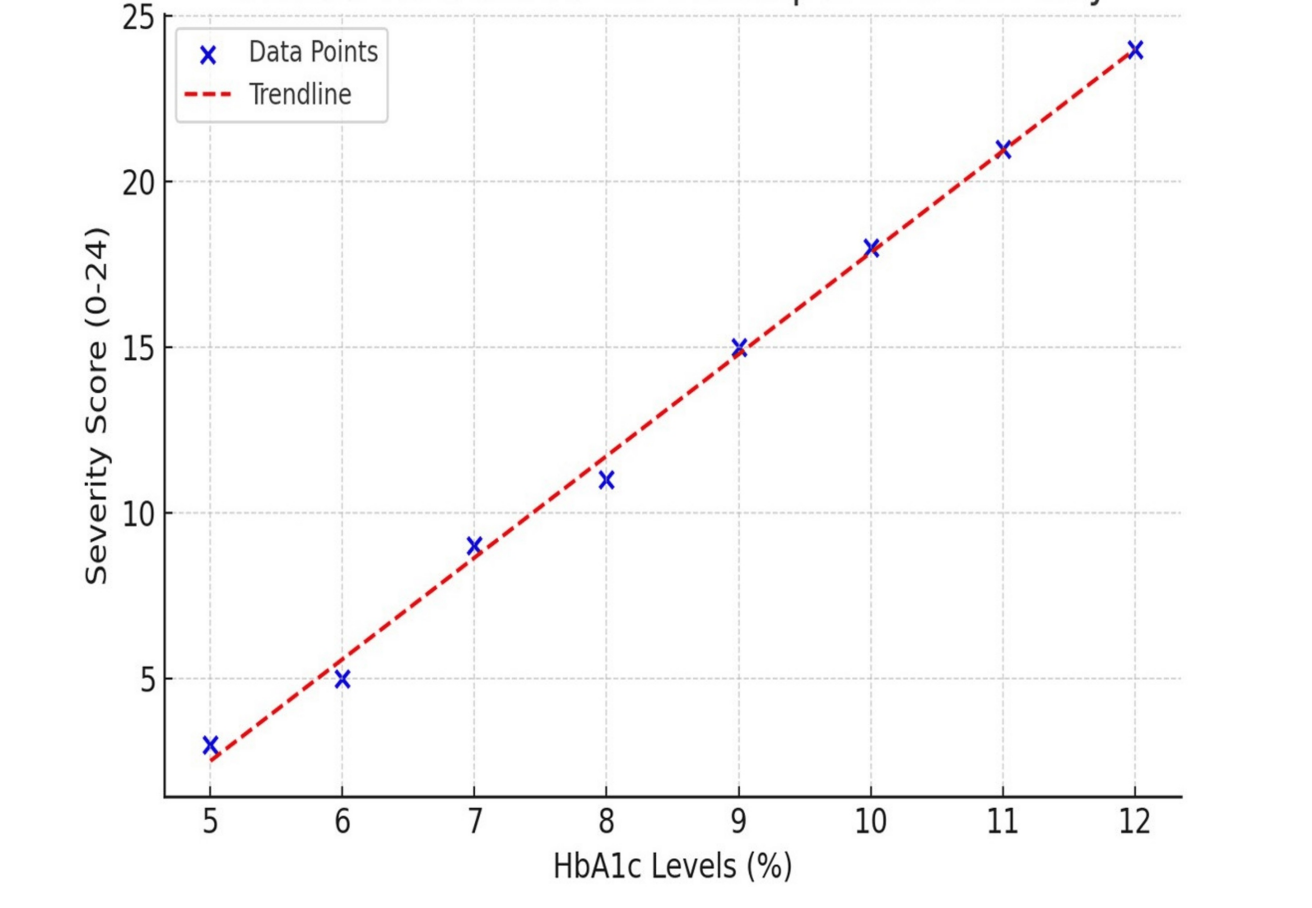
Scatter plot: HbA1c vs balanoposthitis severity

The statistical analysis confirmed a significant association between HbA1c levels and disease severity (Figures 2, 3). A strong positive correlation was established between the continuous HbA1c value and the total severity score, with a Pearson's correlation coefficient (r) of 0.45 (p < 0.01). This finding quantitatively supports the hypothesis that progressively higher HbA1c levels are directly associated with increasingly severe clinical manifestations of balanoposthitis.

## Discussion

The current study provides quantitative evidence for a strong positive correlation between poor glycemic control (HbA1c) and the severity of balanoposthitis, establishing a statistically significant link (r = 0.45, p < 0.01). This finding is consistent with established literature recognizing diabetes as a primary risk factor for balanoposthitis [4,5,6,13] and extends it by quantifying the dose-dependent relationship between metabolic control and clinical outcome.

Balanoposthitis is frequently linked to Candida infection, especially in diabetic patients, where chronic hyperglycemia in tissues such as the foreskin and glans creates an environment conducive to fungal proliferation [8,9]. Our data reinforce this, demonstrating a sharp rise in severe symptoms, including erythema and edema, as HbA1c increases [14].

A particularly salient finding is the high prevalence of ulceration (75%) and pain in patients with HbA1c ≥ 9%. The progression from simple inflammation to ulceration suggests that severe hyperglycemia compromises local immunity and tissue healing mechanisms [11]. The sustained elevation of blood glucose drives the formation of AGEs, leading to microangiopathy and neuropathy that impair wound healing and increase susceptibility to severe, nonresolving inflammatory lesions [11]. This outcome is especially significant because severe forms of balanoposthitis can lead to complications such as phimosis [10] and, in rare instances, be the initial presentation that prompts a diabetes diagnosis [7]. Our study emphasizes that these severe, complicated presentations are overwhelmingly concentrated in the group with the poorest long-term glycemic management.

The necessity of integrating endocrinology and dermatology is strongly highlighted by these results. Although topical antifungal or anti-inflammatory agents are the mainstay of balanoposthitis treatment [1], our findings suggest that these interventions alone will likely fail in patients with poor HbA1c control. Effective management must fundamentally address the underlying metabolic derangement [6]. A multidisciplinary approach that ensures aggressive glycemic control is initiated concurrently with specific topical treatments is paramount to achieving clinical remission and preventing recurrence or progression to chronic complications [2,15].

Limitations and future scope

The study's cross-sectional design captures a single point in time, preventing the establishment of definitive causality or the tracking of disease progression. Although the sample size of 50 patients provided sufficient power to demonstrate a significant correlation, caution should be exercised in generalizing the findings. Other confounding factors, such as the specific duration of diabetes in each patient or the impact of concurrent unmeasured immune factors, were not fully accounted for. Future research should prioritize larger longitudinal studies that follow patients from diagnosis, correlate weekly or monthly glucose fluctuations with symptom flares, and specifically evaluate the impact of lowering HbA1c on the subsequent reduction of severity scores.

## Conclusions

The findings of the "Glycemic Gravity" study clearly establish a strong positive correlation (r = 0.45, p < 0.01) between elevated HbA1c levels and increased balanoposthitis severity in diabetic males. This study quantitatively confirms that poor glycemic control is the primary metabolic driver of disease severity, leading to more pronounced inflammation, fissuring, and higher pain scores. Beyond immediate symptom management, this correlation carries significant long-term prognostic implications. Sustained high-severity inflammation due to uncontrolled diabetes is a known precursor to irreversible fibrotic complications such as secondary phimosis and balanitis xerotica obliterans (BXO). Furthermore, our data indirectly reinforce the urgency of treatment, given the documented association between chronic penile inflammation and an elevated risk of penile carcinoma. Therefore, the management paradigm for diabetic balanoposthitis must fundamentally shift from a purely topical approach to an integrated multidisciplinary care model.

A clinical imperative exists in which patients presenting with a high balanoposthitis severity score (e.g., above a clinical threshold of 15 on the 0-24 scale) should be triaged promptly for co-management, prioritizing aggressive glycemic optimization by an endocrinologist alongside local dermatological therapy. A preventive strategy should recognize early and effective diabetes management as the most powerful measure against severe, recurrent, and complicated balanoposthitis. A future direction for research involves longitudinal studies to explore whether intensive glycemic correction, meaning a reduction in HbA1c, leads to a demonstrable and quantifiable reduction in the balanoposthitis severity score over time. In essence, the severity of local inflammatory disease of the penis serves as a visible biomarker for systemic metabolic failure. By integrating dermatology and endocrinology, clinicians can not only improve the local condition but also significantly reduce the patient's long-term morbidity risk.
